# Effects of Chitin and Contact Insecticide Complexes on Rove Beetles in Commercial Orchards

**DOI:** 10.1673/031.011.9301

**Published:** 2011-07-25

**Authors:** A. Balog, L. Ferencz, T. Hartel

**Affiliations:** ^1^ Greeley Memorial Laboratory, School of Forestry and Environmental Studies, Yale University, 370 Prospect, 06511-New Haven, Connecticut, USA; ^2^ Department of Horticulture, Faculty of Technical Sciences, Sapientia University, I/C Sighişoarei St. Tirgu-Mureş, MS-540485, Romania; ^3^ Mihai Eminescu Trust, 10 Cojocarilor St. Sighisoara 545400, Romania

**Keywords:** abundance, insecticide, physico-chemical properties, species richness, steric energy

## Abstract

A five-year research project was performed to explore the potential effects of contact insecticide applications on the change of abundance and species richness of predatory rove beetles (*Coleoptera: Staphylinidae*) in conventionally managed orchards. Twelve blocks of nine orchards were used for this study in Central Europe. High sensitivity atomic force microscopic examination was carried out for chitin structure analyses as well as computer simulation for steric energy calculation between insecticides and chitin. The species richness of rove beetles in orchards was relatively high after insecticide application. Comparing the mean abundance before and after insecticide application, a higher value was observed before spraying with alphacypermethrin and lambda-cyhalothrin, and a lower value was observed in the cases of diflubenzuron, malathion, lufenuron, and phosalone. The species richness was higher only before chlorpyrifos-methyl application. There was a negative correlation between abundance and stability value of chitin-insecticides, persistence time, and soil absorption coefficients. Positive correlation was observed with lipo- and water solubility.

## Introduction

Staphylinidae is the largest Coleoptera family that includes predators as well. More than 47,000 species are known worldwide, and probably over 75% of tropical species are still undescribed (Howard et al. 1998). Between 2500–2700 species were recorded from Europe, but staphylinid fauna of agricultural landscapes is still little known (Balog et al. 2008), especially the effects of insecticide use on these predators (Krooss and Schaefer 1998; Andersen 1991; Andersen 2000; Perner and Malt 2002; Balog et al. 2009).

For the past twenty years, the Game Conservancy in Sussex, UK has monitored abundances of invertebrates in agricultural fields. The total number of invertebrates recorded per sample dropped by almost half in the course of the present study, corresponding to a quarter of what was present in 1970. This overall change was the result of widespread declines in some common groups such are *Araneae*, *Lepidoptera*, *Aphididae* *(Hemiptera), Symphyta (Hymenoptera), Staphylinidae, Cryptophagidae, Lathridiidae*, and *Lonchopteridae (Diptera)*; these groups constituted 72%, on average, of the total number. The results of the present study showed that the insecticide application has less undesirable effects on rove beetles when compared with other groups. Rove beetles show high abundance and species richness in both conventional and abandoned systems (Aebischer and Potts 1990).

Field experiments compared the sensitivity of rove beetles, ground beetles and spiders to three insecticides (pirimicarb, deltametrin, and dimethoate), and demonstrated that rove beetles presented a higher abundance compared to other groups the cause of this difference was not known (Andersen 1982; Wickerman et al. 1987; Good and Giller 1991). The effects of insecticides on staphylinids' fitness were studied both under laboratory conditions (Samsøe—Petersen 1993, 1995a, 1995b; Botha 1994; Botha and Plessis 1995) and field experiments, in cereal crops (Sunderland 1992; Shah et al. 2003). Mortality, egg production, and fertility were all adversely affected by insecticides. Fungicides and herbicides were less detrimental with few exceptions: tetradifon, classified as harmless; and the fungicides oxythioquinox, thiram, and afugan, rated as moderately harmful. Among growth regulators, carbaryl was found to be highly detrimental, however, to chlormequat chloride and alpha naphtyl acetic acid had no effects on staphylinids. Oxythioquinox, ziram, and diuron strongly affected egg survival (Samsøe-Petersen 1993). As for the urea herbicides, the strongest effect was exerted by methabenzthiazuron that caused total reproductive failure, killing the eggs. Strong negative effects on various life stages were noticed in bromoxynil, pyridate, haloxyfop, and carbaryl (the latter being also used as plant growth regulator) (Samsøe-Petersen 1995a, 1995b; Botha 1994; Botha and Plessis 1995).

Only few studies examine the resistance mechanisms on insects (Chaudhry 1999; Andras et al. 2007; Rimmer et al. 2009) and according to our knowledge no studies address the formation of complexes between chitin and insecticides. The aim of this study was to fill this gap and determine the change in abundance of rove beetles after insecticide applications in conventionally managed orchards. We hypothesize that interactions with chitin may be one way in which insecticide's affect rove beetles tolerance and sensibility.

## Materials and Methods

### Sample collection

Studies were carried out in twelve blocks of nine orchards from 1998 to 2002 in Central Europe, Hungary. Six orchards consisted of one apple block each, while the remaining three orchards had an apple block and a pear block (Table 1).

Five orchards were located on sand and four on clay (Table 1). Neither tillage nor irrigation was used during the experiment. Eleven blocks were treated with registered broad spectrum contact insecticides; one was untreated used as control. On average, 10 treatments (one in every second week) were applied in each block per year from mid-April to mid-October (Table 2).

Ten pitfall traps (covered, 300 cm3 in size, 8 cm in diameter, half-filled with ethylene glycol 30% solution) were placed from the field margin towards the field centre at 10 m intervals within each block. Samples were collected from mid-April until mid-October. The sample collections were synchronized with pesticide applications and completed before and after 2–3 days of insecticide use. The number of caught individuals and species allows a direct estimation of abundance and species richness, considered the average number of individuals and species after each treatment in ten pitfall traps. All staphylinids were sorted and identified up to species level (Freude et al. 1964, 1974; Zerche 1996a, 1996b).

**Table 1.**
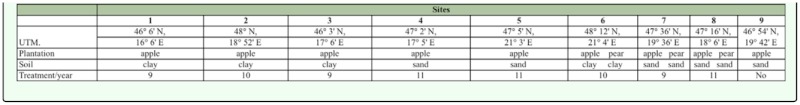
Geographical localization and characteristics of the investigated orchards.

### Chitin analyses and molecular dynamics simulation

The chitin surface from the dorsal abdominal part of all species was analyzed with atomic force microscopic investigation. Beetles were collected at each sampling period (i.e. before and after insecticide application) and analyzed species were caught only after insecticide use. Ethylene glycol did not modify the surface characteristics of the chitin. The sample size for each species-insecticide was 2. The exoskeletons were first split horizontally and then the surfaces of each of all three layers were scanned. Computer simulation and steric energy calculation were performed with molecular modeling software Chem3D Ultra version 10.0 to study complexation processes between contact insecticides and chitin.

Molecular dynamics simulation studies in vacuum were performed at a temperature of 300K (26.8° C) over a period of 100 ps (picoseconds). The molecular dynamics computation consisted of a series of steps, which occurred at fixed intervals, typically about 2.0 fs (femtoseconds). The Beeman algorithm for integrating the equations of motion was used to compute new positions and velocities of each atom at each step. Each atom was moved according to the following formula:
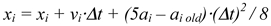


Similarly, each atom was moved for *y* and *z*, where *x*_i_, *y*_i_, and *z*_i_ are the Cartesian coordinates of the atom, *v*_i_ is the velocity, *a*_i_ is the acceleration, *a*_i *old*_ is the acceleration in the previous step, and Δ*t* is the time between the current step and the previous step. The potential energy and derivatives of potential energy (*g*_i_) were then computed with respect to the new Cartesian coordinates. New accelerations and velocities were computed at each step, according to the following formulas (*m*i is the mass of the atom):
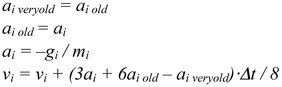


**Table 2.**
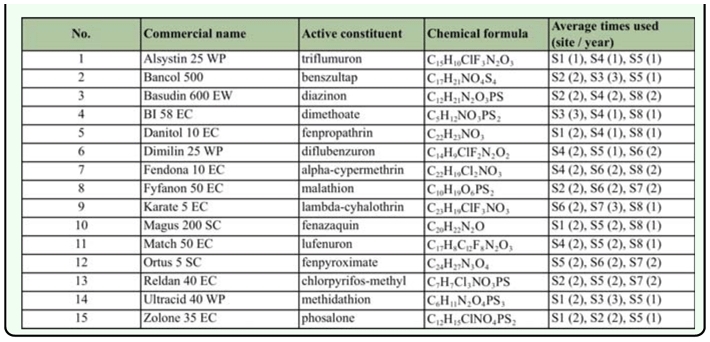
Contact insecticides used in the investigated orchards. S1…S8 — Sites investigated, (1…3) — average number of use the insecticide / year.

Along with conformational parameters, the formation of hydrogen bonds was monitored during the whole simulation time (Ferencz et al. 2006). Finally, the steric energy and the stability indexes (*Ds*) were calculated between chitin and contact insecticides applied during the experiment, using the following formula:
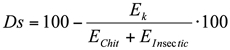

where *Ds* is the stability index and represents the percent decrease of the complexes' steric energy (*E_k_*) compared to the sum of the energy of the host (*E_Chit_*) and guest (*E_Insectic_*) (Ferencz et al., 2006). The complexes with *Ds* > 50% were considered stable, whereas the complexes with *Ds* < 35 unstable.

Analyses of variance were used to compare the mean abundance and species richness/10 trap/assessment for each site of study. Two-way ANOVA were used to compute the means of abundance and species richness before and after insecticide applications and to compare the mean abundances, respectively; species richness after insecticide treatment; and stability index (*Ds*) of the complexes.

Based on results of complexation between chitin and tested insecticides, correlation coefficient was computed between different physico-chemical properties (*Ds*, liposolubility, persistence, water solubility, and soil adsorption coefficient) and abundance after each insecticide using EPI Suite v 3.20 and *Regressim*, a personal developed software (©Ferencz 1997). The equation of the multiple regressions was the following:
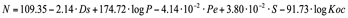


## Results

### Sample collection

Altogether, 6187 individuals belonging to 238 species were collected. The most frequently found were *Dinaraea angustula* (Gyllenhal, 1810), *Palporus nitidulus* (Fabricius, 1781), *Aleochara bipustulata* (L., 1761), *Oligota pumilio* (Kiesenwetter, 1858), *Dexiogyia corticina* (Erichson, 1837), *Xantholinus linearis* (Olivier, 1794), *Xantholinus longiventris* (Heer, 1839), *Sphenoma abdominale* (Mannerheim, 1830), and *Omalium caesum* (Gravenhorst, 1806). These accounted for 45.54% of the total individuals in the twelve experimental sites. Significant differences in abundance were observed for all sites, except Site 1. The species richness was low in Site 5 and high in the control plot (Table 3).

### Chitin analyses and molecular dynamics simulation

**Figure 1.**
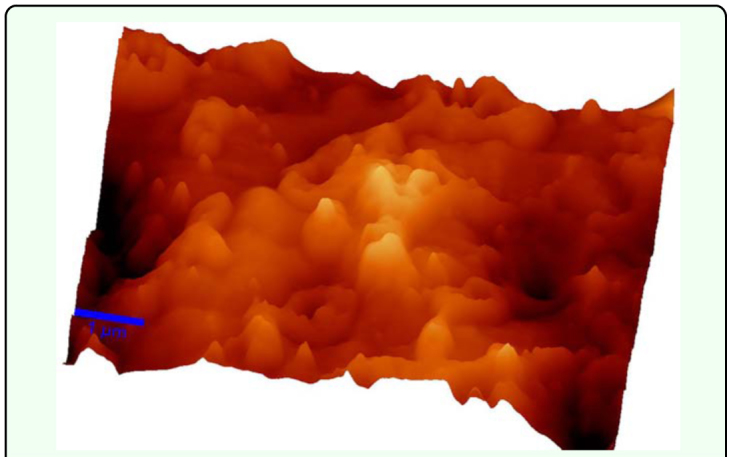
The atomic force microscopic image of the chitin surface of a rove beetle. High quality figures are available online.

Atomic force microscopic analyses revealed that the surface layers of the chitin were relatively similar for each species; all of them being extremely uneven (rough or rugged) presenting several protuberances and valleys. These structures allow the formation of complexes between chitin and insecticides (Figure 1).

**Table 3.**
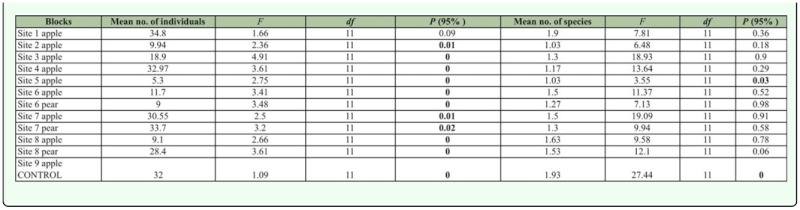
Mean abundance and species richness / 10 trap / assessment for each site of study (ANOVA).

*Ds* value was computed for each complex (chitin + insecticide) (Table 4). The higher the decrease of the steric energy, the more stable the formed complex. Complexes with *Ds* > 50 were considered stable, whereas complexes with *Ds* < 35 were considered unstable. Six insecticides formed a very stable complex with chitin; three were relatively unstable, while the other six produced medium stability, in many cases with *Ds* > 45 (Table 4).

By modeling the complexation process between insecticides and chitin, differences (key-lock type conformational fittings) were recorded not only in insecticide, but also in chitin molecules. Complexes with triflumuron were among the highest because of the polarity of the insecticide molecule, the presence of the F and C1 atoms and amidic groups, which contributed to stable hydrogen and van-der-Waals interactions with chitin. By modeling the formation of complexes between chitin and alpha cypermethrin, high stability (*Ds* = 50.22) was observed due to the polar molecule of cypermethrin. Lambdacyhalothrin seems to form a stable complex with chitin because of the possibility of steric adaptation of the apolar part on the chitin surface; however, this *Ds* value was only 21.82. The apolar molecule of fenazaquine establishes a more unstable complex with the substrate (chitin), which may be due to the extended hydrophobic molecular surface.

**Table 4.**
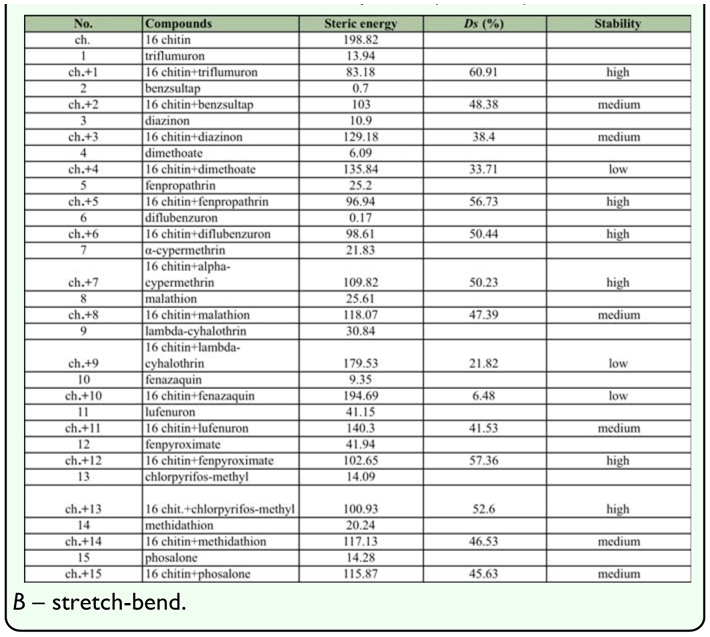
Steric energies, its components and the stability (*Ds*) of the chitin, insecticides and its complexes (kcal/mol).

**Figure 2.**
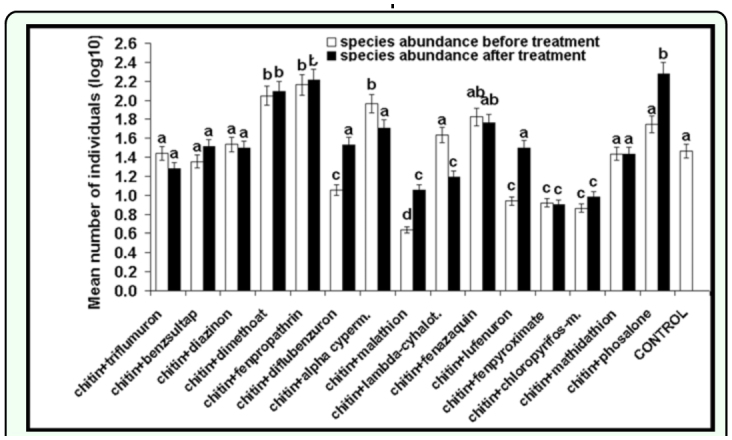
Species abundance (log10) before and after insecticide application (average numbers of individuals in 10 pitfall tap/assessment). (Two-way ANOVA, different letters mean statistical significant differences). High quality figures are available online.

**Figure 3.**
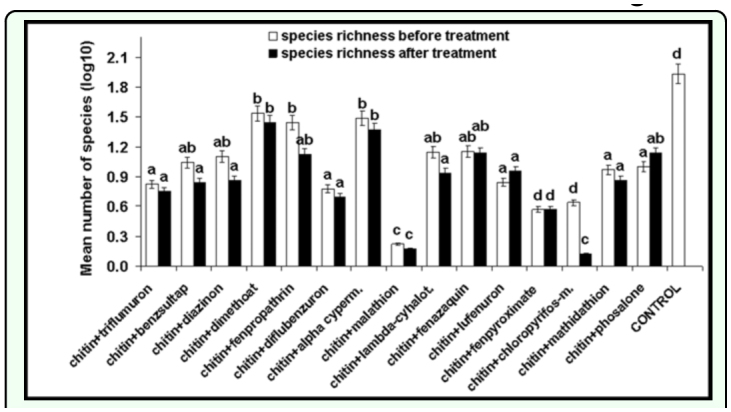
Species richness (log10) before and after insecticide application (average numbers of individuals in 10 pitfall tap/assessment). (Two-way ANOVA, different letters mean statistical significant differences). High quality figures are available online.

The cumulative species abundance was similar before and after insecticide applications (*F* = 1.21, *df* = 11, *P* < 0.24), however the cumulative species richness was higher before treatments (*F* = 2.77, *df* = 11, *P* < 0.01). Comparing the mean abundance before and after insecticide application individually for each insecticide, a higher value was observed before alphacypermethrin (*Ds* = 50.23) (*F* = 2.41, *df* = 14, *P* < 0.03) and lambda-cyhalothrin (*Ds* = 21.82) (*F* = 1.31 , *df* = 14, *P* < 0.01) spraying and lower abundance before diflubenzuron (*Ds* = 50.44) (*F* = 2.76, *df* = 14, *P* < 0.01), malathion (*Ds* = 47.39) (*F* = 2.91, *df* = 14, *P* < 0.01, lufenuron (*Ds* = 41.53) (*F* = 2.10 , *df* = *14, P* < 0.05), and phosalone (*Ds* = 45.63) (*F* =2.40 , *df* = 14, *P* < 0.03) application (Figure 2). The species richness was higher before chlorpyrifos-methyl (*Ds* = 52.59) (*F* = 1.20, *df* = 14, *P* < 0.001) use (Figure 3).

## Discussion

The findings are consistent with other studies, which also showed high abundance and species richness of rove beetles in conventional systems (Shah et al. 2003; Weisberg and Reisman 2008).

In other experiments, the chlorpyrifos-methyl toxicity was 23.5 times lower, whereas the malathion toxicity was 32.0 times lower after 24 h (Kljajić and Perić 2006). Other authors found dichlorvos to have the highest mean lethal concentration on beetles, *Sitophilus oryzae* (L.) (*Coleoptera: Curculionidae*), after 4–5 hours of contact (Champ et al. 1969; Grenier and Grenier 2008). Another study reported chlorpyrifos-methyl toxicity was 1.6 times higher than malathion after 7 hours of exposure of normally susceptible *S. oryzae* weevils, and toxicity to *S. granarius* was 6.5 times higher. Chlorpyrifos-methyl was 3.5 times more toxic to granary weevils than to rice weevils, while malathion showed similar toxicity to both species (Williams and Guesclin 1978; Relyea 2005).

**Table 5.**
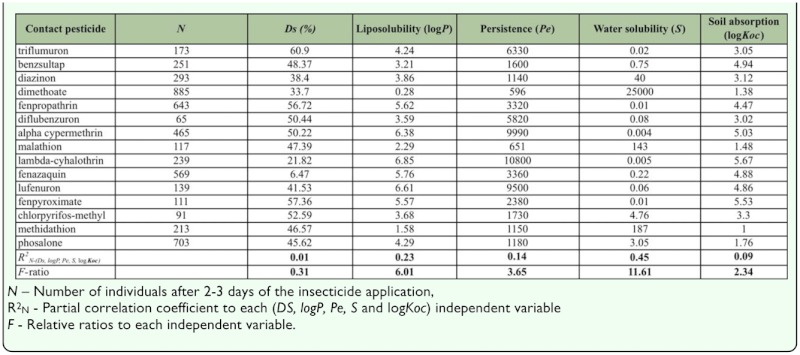
The partial correlation coefficient between the physico-chemical properties of the insecticides and the abundance of rove beetles after 2–3 days of insecticide application.

According to these results, insecticides which form stable complexes with chitin have longer persistence on the insect body, and the probability of penetration of active component through the chitin to the target site is slowed down. Species abundance did not change in most of the cases, but in some cases it increased (for diflubenzuron, malathion, lufenuron, and phosalone) after insecticide treatment. The species richness, however, remained the same or decreased (for chlorpyrifos-methyl) after the application of the same insecticides. Few studies reported the insecticide resistance of rove beetles, and no studies address the molecular background of resistance for this group. In a greenhouse evaluation of the effects of pesticides, when releases of *A. coriaria* adults were performed both before and after application of the designated pesticide solutions, both chlorpyrifos at both low and high label rates and chlorfenpyr were directly harmful to the rove beetle, *A. coriaria. Bacillus thuringiensis* subsp. *israelensis*, flonicamid, spinosad, and azadirachtin, however, were not directly toxic to *A. coriaria* adults (Cloyd et al. 2009). In open field conditions such as apple orchards, the greater mobility of staphylinids, which fly more readily than the most Coleoptera, may enable them to avoid pesticide applications in individual fields; but it may also enable them to quickly re-colonize orchards. However, considering these factors together they cannot fully explain the fluctuations in density immediately after insecticide application. Therefore, based on our results, the correlation coefficients between different physico-chemical properties of the insecticides such as stability (*Ds*), liposolubility or octanol - water partition coefficient (log*P*), persistence in hour (*Pe*), water solubility in mg/L (*S*), soil adsorption coefficient (log*Koc*), and the average density of rove beetles after 48 h of insecticide application (*N*) (Table 5) were computed. A negative correlation was observed between stability (*Ds*), persistence time (*Pe*), soil absorption coefficient (log*Koc*), and abundance. As these values increased, the abundance decreased. The relation was weak only in the case of fenpropatrin, due to a species of *Dinaraea angustula* (Gyllenhal 1810), which accounted for more than 50% of the total individuals in only one of the orchards. Positive correlations were observed between the following parameters: octanol —water partition coefficient (log*P*), water solubility (S) (mg/L), and abundance (Table 5). All in all, the multiple correlation coefficient (*R*^2^) was 0.6492 (*R* — 0.8057, *F* — 3.33), which means that these factors together determined 80.57% the abundance of rove beetle species 2–3 days after insecticide treatment.

We can conclude that certain contact pesticides can be compatible or partially compatible with, and can be used along with, rove beetles species in apple systems that use these natural enemies to manage insect pests. Further studies are needed to fully understand the physiological background of conventional chemical—use on beneficial insects in agricultural fields.
